# It all started with a sore throat: Polymicrobial septicaemia, cavitating lung lesions and severe thrombocytopenia

**DOI:** 10.1016/j.clinme.2024.100260

**Published:** 2024-10-26

**Authors:** Edmund Hugh Larkin, Ana Garcia-Mingo, Roopal Patel, Andrew Badacsonyi

**Affiliations:** a Intensive Care Unit, Whittington Health Trust, London, United Kingdom; b Infectious Diseases and Microbiology, Whittington Health Trust, London, United Kingdom; c Radiology, Whittington Health Trust, London, United Kingdom

**Keywords:** Lemierre's syndrome, *Fusobacterium necrophorum*, Thrombophlebitis of the internal jugular vein, Cavitating lung lesions, Thrombocytopenia

## Abstract

We report a case of a syndrome first described by French bacteriologist André Lemierre nearly 100 years ago. A young woman presented with fever, chest pain and arthralgia. Admission investigations revealed thrombocytopenia, cavitating pneumonia and an internal jugular vein thrombus. Blood cultures were positive for *Fusobacterium necrophorum* and *Streptococcus constellatus.* Respiratory failure developed within 48 h and was managed with high-flow nasal oxygen in a critical care setting. The investigation findings leading to diagnosis, as well as the antimicrobial and anticoagulation management strategies, are discussed. Lemierre's syndrome is rarely encountered by non-specialists, but a good outcome can be expected with prompt diagnosis and treatment.

## Highlights

• Lemierre's syndrome is a life-threatening, systemic complication of, in most patients, pharyngitis or tonsillitis.
• It should also be considered in young adult patients with a sepsis-type presentation and cavitating lung lesions or multiple pulmonary emboli.
• Thoracic and neck vessel imaging is of critical diagnostic value in suspected patients, particularly in the absence of an identified bacteraemia or prior to sensitivity results.
• The relationship between *Fusobacterium* infection and thrombocytopenia remains unclear, and conclusive evidence is needed to provide guidance on the role of anticoagulation in Lemierre's syndrome.
• Full recovery can be anticipated following diagnosis and successful antimicrobial management.

## Background

The clinical syndrome originally described by Lemierre is of anaerobic bacterial pharyngotonsillitis and associated septic thrombophlebitis of the internal jugular vein.^1^^,^^2^ There are no formally agreed diagnostic criteria. The most common causative anaerobic organism is *Fusobacterium necrophorum*, which is reported to have been directly implicated in over 80% of cases.^3^ Other associated organisms include *Streptococcus* and *Staphylococcus*. Polymicrobial infection and culture of methicillin-resistant *Staphylococcus aureus* is also described.^4^

One broad group of *Fusobacterium* spp. infections occur as a result of peri-tonsillar abscess or thrombus invasion into local structures of the head and neck. However, classical or metastatic Lemierre's refers to disseminated infection, which arises following thromboembolism and abscess formation at distant sites. The most common example of this is septic pulmonary emboli and associated cavitating lung lesions, in 70–97% of cases.^5^^,^^6^ Less frequent other sites of embolic dissemination are the joints, muscle, bone and liver.^7^ A core component of *F. necrophorum* virulence is its propensity for clot formation: although this is likely multifactorial, *F. necrophorum* can induce platelet aggregation directly, and this is thought to be driven by haemagglutinin present on the bacterium cell surface.^8^ One recent review suggests that a positive culture result for *Fusobacterium* spp., alongside the presence of distant septic emboli, are as useful in diagnosis as radiological evidence of internal jugular vein thrombosis.^9^

## Case presentation

A previously healthy female in her 20s presented with 1 day of confusion, pleuritic chest pain and a painful left hallux. This had been preceded by a sore throat for 1 week. She had no medical or family history. She smoked five cigarettes per day.

Observations and examination findings in the emergency department were as follows: temperature 38.5°C, respiratory rate 20 breaths per minute, oxygen saturation on room air 95%, blood pressure 110/60 mmHg and heart rate 115 beats per minute. She was disorientated to time and place. Auscultation of the chest revealed reduced air entry at the left base, but was otherwise unremarkable. Heart sounds were normal and capillary refill time was <2 s. The left first MTP joint and left fifth PIP joint of the hand were both erythematous and tender to palpate. There was no rash, meningism, tonsillar exudate, neck swelling, cervical lymphadenopathy or focal neurological signs. Blood results at presentation are detailed in Table 1.

**Table 1:** Blood results at presentation.

Test	Result	Reference range
Hb	116	115–165 g/L
White blood cells	17.7	3.5–12×10^9^/L
Platelets	8	140–400×10^9^/L
Neutrophils	16.4	1.7–7.5×10^9^/L
Lymphocytes	0.7	1–4×10^9^/L
C-reactive protein	220	0–5 mg/L
Sodium	121	135–145 mmol/L
Potassium	4.6	3.5–5.1 mmol/L
Urea	13.9	2.1–7.1 mmol/L
Creatinine	126	49–92 mmol/L
Bilirubin	22	0–21 µmol/L
ALT	33	0–33 iu/L
Albumin	30	35–50 g/L
INR	1.3	0.8–1.3
PTT	56.4	25–38 s
PTT ratio	1.7	0.8–1.2
Derived fibrinogen	>500	180–400 mg/dL

**Fig. 1:**
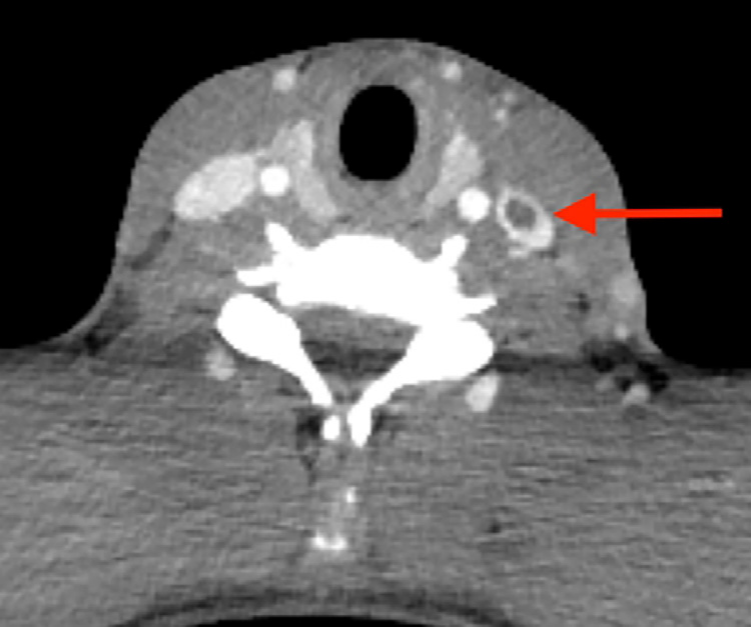
CT pulmonary angiogram mediastinal window: left internal jugular vein with filling defect, representing a thrombus. Of note, a subsequent CT venogram report also identified mild left-sided tonsilitis and further filling defects in the common facial and external palatine veins.

**Fig. 2:**
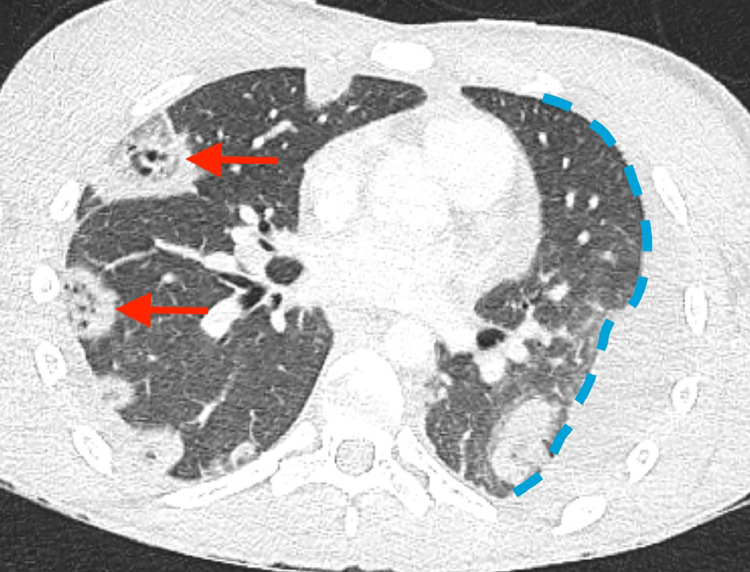
CT pulmonary angiogram lung window: multiple cavitating nodules, positioned both peripherally and centrally. Two of these are highlighted by red arrows. A loculated pleural effusion is also delineated in blue.

While in the emergency department, blood cultures were obtained and intravenous crystalloid resuscitation administered. Immediate antimicrobial treatment was with intravenous co-amoxiclav as broad cover for sepsis of unknown source, while awaiting imaging and culture results. Admission chest X-ray revealed bilateral, scattered opacities in both lung fields, and the patient described worsening pleuritic chest pain. Cross-sectional imaging of the chest was arranged. Within 8 hours of arrival in hospital this had been reported, and suggested internal jugular vein thrombosis and septic thromboembolism (Fig. 1, Fig. 2). At 12 hours, the anaerobic blood culture revealed Gram-negative rods and Gram-positive cocci in chains. Following specialist microbiology advice, intravenous ceftriaxone and metronidazole were initiated. Despite treatment, the patient remained febrile (38–39.5 °C), exhibited worsening tachycardia (140 bpm) and developed type 1 respiratory failure (100 % FiO_2_ via non-rebreathe mask to maintain target saturations). She was admitted to intensive care on day 2 of admission, for high-flow nasal oxygen and invasive blood pressure monitoring. On day 3, blood culture isolates were confirmed as fully sensitive *Streptococcus constellatus* and *Fusobacterium necrophorum* (Table 2). On this basis, treatment with high-dose benzylpenicillin and metronidazole was commenced. Persisting respiratory failure and rising inflammatory markers prompted repeat CT imaging of the chest, which identified increased size of the bilateral loculated pleural effusions. For this reason left- and right-sided chest drains were placed on days 5 and 6, respectively. Pleural fluid samples were consistent with empyema (Table 2). Hypoxia improved and inflammatory markers were halved by day 8.

**Table 2:** Further investigations.

Blood cultures	Aerobic blood culture: Signal detected at 72 h *Streptococcus constellatus* Anaerobic blood culture: Signal detected at 12 h *Fusobacterium necrophorum* and *Streptococcus constellatus* Sensitivities: *S. constellatus* sensitive to penicillin, clindamycin, clarithromycin, tetracycline. *F. necrophorum* sensitive to co-amoxiclav, metronidazole, meropenem.

Throat swab – culture	No beta-haemolytic streptococci (groups A, C or G). No *Fusobacterium* spp.
Pleural fluid	Microscopy and culture: No cells or organisms seen. No growth after 48 h incubation. Cytology report: Turbid yellow fluid. Features consistent with an empyema. Biochemistry: pH 6.92 (7.60–7.64) Glucose <0.1 (3.0–5.6 mmol/L) LDH 13,690 (135–214 IU/L) Fluid protein 37 (10–20 g/L) Serum protein 43 (60–80 g/L)

Ultrasound was performed on both the left first MTP and left fifth PIP joint (Table 3). Although findings suggested possible septic arthritis, the effusion volume was inadequate for joint aspiration.

**Table 3:** Ultrasound report – left hand and foot.

Small echogenic effusion of the first MTP joint and marked hyperaemia of the fifth PIP joint, without effusion.
Possible septic arthritis of the left first MTP and left fifth PIP joints.

The patient was seen in both respiratory and intensive care follow-up clinics at 3 months post-discharge and had made a full recovery. A repeat chest X-ray demonstrated complete resolution of the abnormalities seen while an inpatient, other than a small amount of pleural thickening in the left lower zone.

## Treatment

### Antimicrobial therapy

This patient was initially treated with co-amoxiclav 1.2 g, before changing to ceftriaxone 2 g and metronidazole 500 mg on the basis of the initial Gram stain report. With the cultured organisms and sensitivities confirmed, treatment with benzylpenicillin 2.4 g every 4 hours and metronidazole 500 mg every 8 hours was established. After significant clinical improvement, oral antibiotic therapy was modified to 8-hourly co-amoxiclav 625 mg plus amoxicillin 500 g and she was discharged with advice to complete a total of 6 weeks.

### Anticoagulation

Severe thrombocytopenia at presentation prompted transfusion with a single pool of platelets. After the platelet count increment to >50 on day 3, the decision was made to treat the multiple emboli with treatment dose tinzaparin at initial dose 44 units/kg twice daily. After the platelet count normalised, the dose was increased to 175 units/kg once daily, until discharge. At this point, rivaroxaban 20 mg once daily was commenced for a 3-month period.

### Respiratory support

The patient was weaned from high-flow nasal oxygen over a period of 4 days. Bilateral chest drains were inserted and subsequently removed 72 h post-insertion without complication. Note that at the time of insertion, the platelet count and clotting function were normal. Each drained approximately 700 mL of serosanguinous fluid. See Table 2 for pleural fluid sample analysis.

## Discussion

Diagnosis of Lemierre's syndrome requires clinical features to be recognised and radiological or laboratory findings to support. This young adult presented *in extremis*, with a preceding history of sore throat and no prior antibiotic exposure. Despite this, there was no evidence to suggest tonsillitis or thrombophlebitis on clinical examination. Observation on chest CT of a left internal jugular vein filling defect in combination with cavitating lung lesions (located both peripherally and centrally) redirected the differential diagnosis towards disseminated *Fusobacterium* infection.^10^, ^11^ The importance of urgent cross-sectional imaging cannot be understated: it prompted anaerobic cover for *Fusobacterium* spp. within 12 h of admission, which could have been critical to the outcome of this case, particularly given that *Fusobacterium* spp. can be challenging to culture and isolates with beta-lactamase activity have been described.^12^ It is likely that, without this information, antimicrobial management would have prioritised other more common infectious illnesses.

This decision was only later supported by blood culture results, positive for both *Streptococcus constellatus* and *Fusobacterium necrophorum*. Although *S. constellatus* (a member of the *Streptococcus anginosus* group) is associated with both pyogenic and disseminated infections, it is also a commensal oral microorganism.^13^ Furthermore, mixed cultures are not uncommon in Lemierre's syndrome.^7^ This is unsurprising given that tonsillar mucosa is a polymicrobial environment,^14^ and dysregulation in the oral microbiome by one organism may have presented a portal of entry to another.

A further challenge in this case was the marked thrombocytopenia, contradicted by multiple thromboses. A direct link between *Fusobacterium* spp. and thrombocytopenia is not described in the literature. However, a systematic review of Lemierre's cases published in 2023 found that the degree of thrombocytopenia correlated with increased risk of thromboembolic events.^15^ Haematology specialist advice was sought at an early stage, on account of other possible causes for thrombocytopenia. Diagnoses of thrombotic thrombocytopenic purpura and disseminated intravascular coagulation were considered, but excluded on definitive testing. Transthoracic echocardiography did not reveal vegetations and the spleen appearance was normal on CT imaging. It was felt that thrombocytopenia was driven by consumption in the setting of sepsis and multiple pulmonary emboli. Although this patient received anticoagulation and had an excellent outcome, the role for anticoagulation in Lemierre's syndrome is not clear: One meta-analysis reviewed the use of anticoagulation and did not demonstrate a significant difference in mortality or thrombus resolution between the patients who received anticoagulants and those who did not.^16^

This case demonstrates that septic emboli can cause severe respiratory failure in young patients with no pre-existing lung disease or comorbidities. One study of Lemierre's syndrome cases between 2000 and 2017 found the median age to be 21 and the mortality to be 4%.^5^ This is comparable to meningococcal meningitis, which carries a mortality of 4.3 %.^17^ This was a young patient who was critically ill at the time of presentation. CT imaging and specialist microbiology input early into her clinical course were critical not only to reach the diagnosis, but to guide treatment toward a positive outcome.

## Patient consent

Written consent was obtained from the patient.

## CRediT authorship contribution statement

**Edmund Hugh Larkin:** Writing – original draft, Visualization, Conceptualization. **Ana Garcia-Mingo:** Writing – review & editing. **Roopal Patel:** Writing – review & editing. **Andrew Badacsonyi:** Supervision.

## Declaration of competing interest

The authors declare that they have no known competing financial interests or personal relationships that could have appeared to influence the work reported in this paper.

## Appendix Supplementary materials

Image, application 1

Image, application 2
