# Supplementary material for: It all started with a sore throat: Polymicrobial septicaemia, cavitating lung lesions and severe thrombocytopenia
Source: Clin Med (Lond). 2024 Oct 26;24(6):100260. doi: 10.1016/j.clinme.2024.100260 (PMC11582737; doi:10.1016/j.clinme.2024.100260)
Supplement: Supplementary file 1 [file mmc1.docx]

**Response Letter 28/08/2024**

Dear colleagues,

I am very grateful that you have taken the time to read and provide feedback on this manuscript.

Alongside this letter you will find attached (i) an updated ‘clean’ manuscript, and (ii) a further copy of the manuscript with tracked changes.

Listed below are my specific responses to editor and reviewer comments. I hope you will find that I have addressed all of these appropriately.

Kind regards,

Dr E H Larkin - Corresponding Author

**Abstract**

*“Use microbiological names in full when first mentioned.”*

**Apologies, this has been corrected.**

**Background**

*“Although Fusobacterium sp is the most common cause of Lemierre’s, there are reports you can reference of other organisms found in the pharynx and rest of ENT found in cases of the syndrome: anginosus group streps as found plus staph aureus, Group A strep, MRSA. Treatment duration is long and identification of organism in pus, throat swab, blood stream is important to correctly tailor antibiotics.”*

**I have added two sentences on other associated organisms, also specifically mentioning that polymicrobial infection and MRSA infection are described in the literature. This is referenced.**

*“The pathogenesis is interesting and worthy of comment to explain the process from upper respiratory tract infection to infected DVT to septic emboli and complications thereof. Why is Fusobacterium particularly prone to this process?”*

**Thank you, I have added two sentences on the relationship between *F. necrophorum* and thrombosis. A reference is included.**

**Case report**

*“It is stated fully sensitive S. constellatus and F. necrophorum in text but in table 2 you don't detail the sensitivities. If penicillin sensitive then later text cautioning about first line CAP URTI antibiotics is less impactful but you could reference there are increasing Fusi isolates that produce beta lactamases so worth covering fusi and streps with a penicillin plus beta-lactamase inhibitor plus/minus metronidazole in clinical Lemierre’s pending cultures.”*

**I have amended Table 2 to include the culture and sensitivity findings for the blood cultures.**

**Addressing the beta-lactamase-producing Fusobacterium; I have added two sentences with a reference in the first paragraph of the ‘Discussion’ section.** *“It would be nice to have supportive evidence regarding infectivity of the pleural effusions, protein, pH, LDH- were these effusions reactive, infective or even transudative from sepsis and hypoalbuminemia. It is perhaps unsurprising they were culture negative given they were drained on antibiotic but there may be investigations that support the case for these being purulent and henceforth making a case for infective chest complications, drainage plus long course antibiotic. If low pH, raised protein, LDH that could perhaps be included in table 2. Presume 16S PCR wasn't sent?
Did the effusions enhance with contrast on CT chest?”*

**The appearance of both effusions loculated. pH was < 7 from fluid sent in both drains. Protein and LDH were also both raised. I added a row with this information in Table 2, alongside cytology reports which were also consistent with empyema. Unfortunately 16S rRNA PCR was not sent and the cultured fluid was negative.**

*“Did the effusion drainage cause improvement in sepsis features or just respiratory function?, the former also suggesting source control of an infective process?”*

**On the day of chest drain insertion, the patient had been severely hypoxic and was close to requiring intubation. Although there had been a significant fall in inflammatory markers initially, WC and CRP had plateaued at this stage.**

**The drain achieved a marked improvement in hypoxia (stepped down to 1L via NC 48hrs later) and further biochemical improvement (CRP static ~150 => 40 again, 48hrs later). I have amended paragraph 3 in the ‘Case Report’ section to highlight this.**

*“Table 2 is titled microbiology but there is some radiology here too, perhaps that should be presented clearer as a separate finding.
Table 2- the time to positivity presentation could be clearer”*

**Thank you, I have amended Table 2 to make microbiology results easier to interpret. US reports are now in a separate Table (Table 3).** *“Joint effusions- presume you were unable to tap them for diagnostic microbiology? I would state that.*

**This is now detailed in Table 3 and the penultimate paragraph of the ‘Case Report’ section.**

**Discussion**

*“Line 1, I don’t think this case has an unusual presentation I would drop the word unusual. It’s fairly classic. Perhaps small joint rather than large joint involvement and the dramatic thrombocytopenia are less common but the point this patient has had recent sore throat, is young, no prior antibiotic (worth a mention if true, as i am assuming this), has features of sepsis with IJV DVT and cavitating lung lesions is fairly classic.”*

**Thank you. I have amended the Discussion’s first paragraph to reflect this, including the addition of a sentence on penicillin resistance in Fusobacterium infection.** *“There is a slight lean at the beginning of the discussion and conclusion on early clinical diagnosis prompting empirical anaerobic antibiotic cover, covering anaerobes being 'critical to the outcome'. This statement isn't incorrect especially when fusobacterium is classically slow to grow but there is a good chance empirical URTI/LRTI antibiotics do offer something, many of these bugs are amoxicillin or co-amoxiclav sensitive. Infection management is generally more than the right antibiotic and this case illustrates that well.
Early consideration of diagnosis also prompts investigation for metastatic complications, drainage of pus, joint washout and extended antibiotic treatment of septic DVT as well as consideration of broader spectrum antibiotic use. This comes and also in the concluding point and all elements are critical in the successful clinical outcome.”*

**Again, many thanks – the most exciting aspect of this case was the constellation of typical features, all of which became apparent over the course of a few hours, and which led to a diagnosis.**

**I have amended the Discussion and Conclusion sections slightly to reflect this – that in actual fact Lemierre’s syndrome is a diagnosis can be made based on features of sepsis, IJV thrombosis and pulmonary cavitation, even prior to a positive associated bacteraemia, or being able to obtain a history from a critically unwell patient.**

**Recognising the diagnosis, allowed us to link infection and pulmonary embolism in a unified diagnosis and highlighted the potential risk of respiratory failure and empyema formation.** *“The marked thrombocytopenia is interesting and unusual- I would expand on the haematology insights here. Was endocarditis excluded? splenomegaly?”*

**Agreed – this was discussed at length during the patient’s admission!**

**Fusobacterium has been shown to aggregate platelets in a lab setting, and has a well-described propensity to cause suppurative thromboses. Having said this, I have been unable to find a definitive causal link between Fusobacterium and thrombocytopenia.**

**A review that I’ve referenced (15) suggests that Lemierre’s with multiple PEs may drive severe thrombocytopenia, however consumptive thrombocytopenia can be caused by sepsis, DIC or pulmonary emboli alone.**

**Regarding infective endocarditis and splenic sequestration - I have added two sentences on this in the Discussion, in paragraph three. This includes a comment on echocardiography and spleen imaging results.**
