# Supplementary material for: It all started with a sore throat: Polymicrobial septicaemia, cavitating lung lesions and severe thrombocytopenia
Source: Clin Med (Lond). 2024 Oct 26;24(6):100260. doi: 10.1016/j.clinme.2024.100260 (PMC11582737; doi:10.1016/j.clinme.2024.100260)
Supplement: Supplementary file 2 [file mmc2.pdf]

BMJ

Consent form

For a patient's consent to publication of images and/or information about them in BMJ publications.

Name of patient: ALANNAH HARTLEY

Relationship to patient (if patient not signing this form):

*Description of the photo, image, text or other material (**Material**) about the patient. **A copy of the Material must be attached to this form:*** CT CHEST, CT NECK VENOGRAM } March 2023

Provisional title of article in which Material will be included: SIGNIFICANT COMPLICATIONS OF LEMIERRE'S SYNDROME IN A YOUNG ADULT

CONSENT

I ALANNAH EVE HARTLEY *[PRINT FULL NAME]* give my consent for the Material about me/the patient to appear in a BMJ publication.

I confirm that I: *(please tick boxes to confirm)*

- ☑ ***have seen the photo, image, text or other material about me/the patient***
- ☑ ***have read the article to be submitted to BMJ***
- ☑ ***am legally entitled to give this consent.***

I understand the following:

(1) The Material will be published without my/the patient's name attached, however I understand that complete anonymity cannot be guaranteed. It is possible that somebody somewhere - for example, somebody who looked after me/the patient or a relative - may recognise me/the patient.

(2) The Material may show or include details of my/the patient's medical condition or injury and any prognosis, treatment or surgery that I have/the patient has, had or may have in the future.

(3) The article may be published in a journal which is distributed worldwide. BMJ's publications go mainly to doctors and other healthcare professionals but are also seen by many others including academics, students and journalists.

(4) The article, including the Material, may be the subject of a press release, and may be linked to from social media and/or used in other promotional activities. Once published, the article will be placed on a BMJ website and may also be available on other websites.

(5) The text of the article will be edited for style, grammar and consistency before publication.

(6) I/the patient will not receive any financial benefit from publication of the article.

(7) The article may also be used in full or in part in other publications and products published by BMJ and/or by other publishers. This includes publication in English and in translation, in print, in digital formats, and in any other formats that may be used by BMJ or other publishers now and in the future. The article may appear in local editions of journals or other publications, published in the UK and overseas.

(8) I can revoke my consent at any time before publication, but once the article has been committed to publication ("gone to press") it will not be possible to revoke the consent.

(9) This consent form will be retained securely and in confidence by BMJ in accordance with the law, for no longer than necessary. Personal data provided in this form will be used and retained in accordance with BMJ's Privacy Policy available at https://www.bmj.com/company/your-privacy/.

Please tick box to confirm the following:

☑ Where this consent relates to an article in *BMJ Case Reports*, I have/the patient has had the opportunity to comment on the article and I am satisfied that the comments, if any, have been reflected in the article.

Signed: Alannah Hartley
Print name: ALANNAH EVE HARTLEY
Address: 80 HIGH STREET N8 7NU, FLAT 4
Email address: alannahevehartley@gmail.com
Telephone no: 07935 569940

If signing on behalf of the patient, please give the reason why the patient can't consent for themselves (e.g. patient is under 18 or has cognitive or intellectual impairment).

Date: 10/1/24

☐ *If you are signing for a family or other group, please tick the box to confirm that all relevant members of the family or group have been informed.*

If the patient is under the age of 18 but has sufficient understanding of the consent process and its implications, they must also confirm their agreement:

Signed:
Print name:
Date of birth:
Date:

Details of person who has explained and administered the form to the patient or their representative (e.g. the corresponding author or other person who has the authority to obtain consent).

Signed:
Print name: DR E. H. LORKIN
Position: INTERNAL MEDICINE DOCTOR
Address: WHITTINGTON HOSPITAL MAGDALA AVENUE N19 5NF
Institution: WHITTINGTON HEALTH
Email address: EDMUND.LARKIN1@NHS.NET
Telephone no: 07557878443 / 02072723070
Date: 06.01.2024
